# DIA-based serum proteomics of the complement–coagulation cascade identifies candidate biomarkers for myasthenia gravis

**DOI:** 10.3389/fnins.2026.1810549

**Published:** 2026-07-13

**Authors:** Zhiguo Lv, Yibin Zhang, Tianrui Shao, Baitong Wang, Qi Lu, Jian Wang, Hanying Xu, Tong Wu, Kaichen Wang, Haorui Shi, Meijin Song, Jianan Chen, Lei Wu, Xinchen Ji, DongXu Li, Jixiang Ren, Jing Lu

**Affiliations:** 1 College of Traditional Chinese Medicine, Changchun University of Chinese Medicine, Changchun, Jilin, China; 2 Department of Encephalopathy, The Affiliated Hospital of Changchun University of Chinese Medicine, Changchun, Jilin, China; 3 Dingxi Hospital Affiliated to Changchun University of Chinese Medicine, Dingxi, Gansu, China; 4 The Third Affiliated Clinical Hospital of Changchun University of Chinese Medicine, Changchun, Jilin, China; 5 Department of Rehabilitation, The Affiliated Hospital of Changchun University of Chinese Medicine, Changchun, Jilin, China; 6 Affiliated Hospital of Changchun University of Chinese Medicine, College of Traditional Chinese Medicine, Changchun University of Chinese Medicine, Changchun, Jilin, China; 7 Research Center of Traditional Chinese Medicine, The Affiliated Hospital of Changchun University of Chinese Medicine, Changchun, Jilin, China

**Keywords:** biomarkers, complement coagulation cascade, cytoskeleton, myasthenia gravis, proteomics

## Abstract

**Objective:**

This study aims to analyze serum protein changes in patients with myasthenia gravis (MG) via proteomics to identify and validate potential biomarkers.

**Methods:**

Serum samples were collected from 10 MG patients and 10 healthy controls. The data-independent acquisition (DIA) quantitative proteomics technique was used to measure serum protein levels and identify differentially expressed proteins. Functional enrichment analyses, including Gene Ontology (GO), Kyoto Encyclopedia of Genes and Genomes (KEGG), and gene set enrichment analysis (GSEA), were performed to determine the enrichment trends of the differentially expressed proteins. On the basis of the *p* values from the enrichment analysis, hierarchical clustering was used to identify significantly enriched pathways in each group. The STRING database was used to construct protein–protein interaction networks of the differentially expressed proteins, identifying the top 50 most closely interacting protein subnetworks and their associated pathways. The target proteins were then validated via mass spectrometry-based targeted proteomics (PRM). Receiver operating characteristic (ROC) curves were plotted for visual analysis. Additionally, serum samples from 20 MG patients and 20 healthy controls (HCs) were collected to validate the proteomic results by enzyme-linked immunosorbent assay (ELISA).

**Results:**

A total of 220 proteins met the exploratory nominal differential-expression criteria between the MG and healthy control groups, including 49 upregulated and 171 downregulated proteins. After Benjamini–Hochberg FDR correction, 138 proteins remained statistically significant, including 23 upregulated and 115 downregulated proteins. Pathway analysis suggested enrichment of complement/coagulation cascades, tight junctions, regulation of the actin cytoskeleton, and Rap1 signaling. Five prioritized proteins—PROS1, PROC, C4BPA, PFN1, and TLN1—were further evaluated by PRM and ELISA. PRM supported their differential abundance and pathway-level association with complement/coagulation regulation and cytoskeleton-related processes, but did not demonstrate a causal damage cascade. ROC analysis showed AUC values greater than 0.8 in this exploratory cohort; however, these estimates should be interpreted cautiously because of the limited sample size. ELISA validation further supported increased PROS1, PROC, and C4BPA levels and decreased PFN1 and TLN1 levels in MG patients compared with controls.

**Conclusion:**

PROS1, PROC, C4BPA, PFN1, and TLN1 represent exploratory candidate serum biomarkers for AChR-positive MG and require validation in larger, multicenter cohorts before clinical application.

## Introduction

Myasthenia gravis (MG) is a chronic autoimmune neuromuscular disorder characterized by progressive skeletal muscle weakness, particularly affecting muscles that control eye movements, facial expressions, chewing, swallowing, and respiration (Meriggioli and Sanders, 2009). The underlying pathophysiology of MG is driven primarily by impaired neuromuscular junction (NMJ) transmission, which results from the presence of autoantibodies that target proteins crucial for synaptic function (Querol and Illa, 2013). The most well-established target of these autoantibodies is the acetylcholine receptor (AChR) on the postsynaptic membrane of the NMJ, although other targets, such as muscle-specific kinase (MuSK) and low-density lipoprotein receptor-related protein 4 (LRP4), are also implicated (Masi and O'Connor, 2022). The disruption of AChR function leads to reduced neuromuscular transmission, muscle weakness, and fatigue—the hallmark symptoms of the disease (Koneczny and Herbst, 2019).

The diagnosis of MG is primarily clinical and is based on characteristic symptoms such as fluctuating skeletal muscle weakness and the demonstration of fatigable weakness during neurological examination. Electrophysiological assessments, including repetitive nerve stimulation and single-fiber electromyography, provide objective supportive evidence. Serological testing for autoantibodies—particularly against nicotinic AChRs—is considered the first-line laboratory investigation (Rousseff, 2021), as approximately 85% of patients with autoimmune MG test positive for AChR antibodies (Vincent, 2002). In the remaining patients, autoantibodies directed against other NMJ components, such as MuSK or LRP4, can be identified (Hoch et al., 2001; Zisimopoulou et al., 2014). However, a small subset of patients lacks detectable autoantibodies against any known antigenic targets and are therefore classified as having seronegative MG. The existence of this subgroup underscores the immunological heterogeneity of MG and highlights the need for more sensitive diagnostic tools and a deeper understanding of the underlying pathophysiological mechanisms (Lazaridis and Tzartos, 2020; Lenti et al., 2022). In these patients, other autoantibodies, including those against MuSK or LRP4 or even unknown targets, may play significant roles in disrupting NMJ and eliciting autoimmune responses (Fichtner et al., 2020). Immune-mediated damage to the NMJ in the MG not only impairs synaptic signaling but also triggers chronic inflammation, leading to the release of cytokines and immune cells that exacerbate tissue damage (Maselli et al., 1991). These processes result in both direct neuromuscular dysfunction and the loss of neuromuscular integrity over time, complicating the disease course. The immunological mechanisms at play in MG are not limited to the production of antibodies against key NMJ proteins. Complement activation and the subsequent formation of membrane attack complexes (MACs) further contribute to damage to the postsynaptic membrane, amplifying the immune response (Michailidou et al., 2025; Howard, 2018). This immune dysregulation may also promote the recruitment of T cells and antigen-presenting cells to the NMJ, where they further perpetuate the inflammatory cycle (Huda, 2023). These complex interactions among autoimmunity, immune cell infiltration, and complement-mediated injury play a central role in the progression of MG (Sanderson, 2022). Despite significant advances in understanding the autoimmune nature of MG, the complete molecular mechanisms remain elusive. The identification of novel biomarkers that can reflect underlying pathogenic processes—such as immune dysregulation, NMJ dysfunction, and inflammation—could offer valuable insights into disease progression and provide more accurate tools for diagnosis, prognosis, and treatment (Huda, 2023).

Recent advancements in proteomics technologies offer promising approaches for revealing complex protein expression profiles in serum and other biological samples in MG (Yang et al., 2023; Lin et al., 2025). Serum proteomics allows for high-throughput analysis of proteins that reflect dynamic changes associated with disease states, enabling the identification of novel biomarkers that could assist in early diagnosis and targeted therapeutic interventions (Birhanu, 2023). By comparing serum proteomic profiles between MG patients and HCs, this study sought to identify differentially abundant proteins and pathway-level alterations associated with MG. Preliminary results suggested altered abundance of several proteins, including PROS1, PROC, C4BPA, PFN1, and TLN1. These proteins were mapped to complement/coagulation regulation, immune-related processes, and cytoskeleton-associated pathways. These findings may provide exploratory clues to systemic immune, complement/coagulation, and cytoskeleton-related changes in MG, but their causal roles and mechanistic connections require further functional validation. Given the complexity of MG pathogenesis—encompassing autoimmune responses, neuromuscular junction dysfunction, and chronic inflammation—a multifaceted research approach is essential. Proteomic analysis offers a powerful tool to decode these intertwined biological processes by identifying disease-specific biomarkers, thereby refining diagnostic accuracy, guiding personalized treatment strategies, and ultimately improving clinical outcomes for patients affected by this debilitating disorder.

## Materials and methods

### Study population and grouping criteria

A total of 30 patients diagnosed with MG and 30 age- and sex-matched healthy controls were enrolled at the Affiliated Hospital of Changchun University of Chinese Medicine between September 2023 and June 2024. Of these, 10 MG patients and 10 HCs recruited between September and December 2023 were included in the proteomic analysis. The remaining 20 MG patients and 20 HCs, enrolled between January and June 2024, constituted the validation cohort for follow-up experiments. All MG patients were diagnosed according to the “Chinese Guidelines for the Diagnosis and Treatment of Myasthenia Gravis (2020 Edition)” (Ting, 2021) and were AChR-positive as confirmed by clinical diagnosis and AChR antibody testing. This study cohort does not include MuSK-positive or seronegative MG patients. The inclusion criteria for MG patients were as follows: (1) the presence of typical clinical features of MG (e.g., fluctuating muscle weakness) and at least one of the following: a positive pharmacological test (e.g., neostigmine test), supportive electrophysiological findings, or seropositivity for MG-related autoantibodies; (2) age between 18 and 75 years; and (3) Myasthenia Gravis Foundation of America (MGFA) clinical classification of classes I–IV. The exclusion criteria for MG patients included the following: (1) a known history of other autoimmune diseases, serious internal medical conditions, malignancies, psychiatric disorders, or MG crisis at the time of screening; (2) a positive pregnancy test, current pregnancy, lactation, or plans for pregnancy during the study period; (3) use of corticosteroids, immunosuppressants, IVIG, plasma exchange, or other MG-specific treatments within 3 months prior to blood collection; and (4) participation in any other clinical study within the preceding 6 months. These criteria were applied to minimize the potential influence of disease severity and treatment history on serum protein expression. Healthy controls were free from autoimmune diseases, malignancies, cardiovascular disorders, diabetes, or other chronic illnesses. The inclusion criteria for the control group were as follows: age ≥18 years and no history of hypertension, diabetes, heart disease, autoimmune disease, or cancer. Individuals were excluded if they were under 18 years old or pregnant at the time of enrollment. All participants provided written informed consent, and the study protocol was approved by The Ethics Committee of the Affiliated Hospital of Changchun University of Chinese Medicine (approval number: CCZYFYLL2022). Clinical variables, including age, sex, disease duration, AChR antibody status, and MGFA clinical classification, were recorded at enrollment. However, because of the limited sample size of the discovery cohort, stratified proteomic analyses and multivariable adjustment according to MGFA class, disease duration, or antibody titer were not performed in the present study.

### Serum sample collection

Serum samples were collected following a standardized protocol. Briefly, peripheral blood was drawn from all participants via serum separator tubes (yellow-top tubes). The collected blood was allowed to clot at 4 °C for 30–60 min. The samples were subsequently centrifuged at 3,000 rpm for 10 min at 4 °C. The supernatant serum was carefully aspirated via a pipette and aliquoted into cryovials. All the serum samples were immediately stored at −80 °C until further analysis.

### Proteomic analysis

#### Protein extraction

Protein extraction was performed by first depleting the most abundant serum proteins using the Pierce™ 14 Abundant Protein Depletion Spin Columns Kit (Thermo Fisher Scientific), according to the manufacturer’s protocol. The low-abundance fractions were collected for subsequent proteomic analyses. Total protein concentration was measured using the Bradford assay, with a standard curve prepared using BSA dilutions. A protein sample of 20 μg was then separated by 12% SDS-PAGE, and protein bands were visualized by Coomassie staining to assess quality and loading consistency.

#### Trypsin digestion

Equal amounts of protein from each sample were used for digestion, and the volume of each sample was normalized with lysis buffer (8 M urea, 1% SDS, and 1 × protease inhibitor cocktail; phosphatase inhibitors were added when required). The protein solution was reduced with dithiothreitol (DTT) to a final concentration of 5 mM at 56 °C for 30 min and then alkylated with iodoacetamide (IAA) to a final concentration of 11 mM for 15 min at room temperature in the dark. Proteins were digested by adding proteinase solution and trypsin, followed by incubation at 37 °C for 4 h and overnight digestion. Peptides were collected by centrifugation at 12,000 × g for 10 min, followed by an additional rinse with deionized water (ddH₂O). The two peptide eluates were combined for downstream analysis.

#### Liquid chromatography–tandem mass spectrometry (LC–MS/MS)

Peptides were resuspended in mobile phase A and separated using an EASY-nLC 1,200 ultrahigh-performance liquid chromatography (UHPLC) system with a home-made analytical column (100 μm i.d. × 25 cm) packed with 1.9 μm/120 Å ReproSil-PurC18 resins. Mobile phase A consisted of 0.1% formic acid and 2% acetonitrile in water, and mobile phase B consisted of 0.1% formic acid and 90% acetonitrile. The elution gradient was set as follows: 7–20% B (0–16 min), 20–32% B (16–24 min), 32–80% B (24–27 min), and 80% B (27–30 min), at a flow rate of 500 nL/min. After separation, peptides were ionized via a nanospray ionization (NSI) source and analyzed on an Orbitrap Exploris 480 mass spectrometer equipped with high-field asymmetric waveform ion mobility spectrometry (FAIMS). The spray voltage was set to 2,300 V, and FAIMS compensation voltages (CVs) were set at −45 V and −70 V. Both precursor and fragment ions were detected in the Orbitrap. The MS1 scan range was 390–810 m/z at a resolution of 30,000. MS2 spectra were acquired at a resolution of 30,000 with a fixed first mass of 200 m/z, using the “define first mass” scan mode. Data-independent acquisition (DIA) was performed using 23 sequential isolation windows, covering a mass range of 390–810 m/z. The average width of the isolation windows is 17 m/z.

#### Proteomic data analysis and differential protein identification

The DIA data were processed via Spectronaut (v17) with the built-in Pulsar search engine and default parameters. Protein identification was performed against the *Homo sapiens* database (Homo_sapiens_9606_SP_20230103.fasta, 20,389 entries), with decoy sequences included to estimate the false discovery rate (FDR). Trypsin specificity allows up to two missed cleavages. Carbamidomethylation of cysteine was set as a fixed modification, whereas methionine oxidation and protein N-terminal acetylation were set as variable modifications. FDR thresholds for protein, peptide, and PSM identification were set at 1%. Changes in protein abundance between groups were evaluated using fold-change analysis and independent-sample t tests. To control for multiple testing, raw *p* values were adjusted using the Benjamini–Hochberg procedure to control the false discovery rate (FDR). Proteins with a fold change > 1.5 or < 1/1.5 and a raw *p* value < 0.05 were considered to meet the exploratory nominal differential-expression criteria. Proteins that also had an FDR-adjusted *q* value < 0.05 were considered FDR-significant differentially expressed proteins. Raw *p* values and FDR-adjusted *q* values for all quantified proteins are provided in Supplementary Table S1.

#### Technical reproducibility assessment

To evaluate the reproducibility and sample consistency of quantitative proteomics, raw protein intensity values were first filtered by masking zero entries as missing values and then log10-transformed. The distribution and variability of protein intensities across samples were assessed using combined violin and box plots. Pairwise Pearson correlation coefficients (PCCs) were calculated across all samples using pairwise complete observations to handle missing values. The exact within-group PCC ranges and median intensity ranges were reported in the Results section.

#### Bioinformatics analysis

Differentially expressed proteins were subjected to Gene Ontology (GO) classification, Kyoto Encyclopedia of Genes and Genomes (KEGG) pathway enrichment, and gene set enrichment analysis (GSEA). Fisher’s exact test was used to calculate the significance (*p* values) of enriched biological functions and pathways. The enrichment results were visualized using bubble plots, Circos plots, and bar graphs.

#### Protein–protein interaction network construction and hub analysis

Differentially expressed proteins with |fold change| > 1.5 were imported into the STRING database^1^ using UniProt IDs (or protein sequences when required), and only interactions with a combined confidence score > 0.7 (high confidence) were retained. For network analysis, a high-confidence PPI network was constructed and node degree (number of interacting partners) was calculated for each protein. STRING’s default Markov clustering algorithm (MCL) was used to identify tightly connected subnetworks (modules). A high-connectivity subnetwork was then generated by selecting the top 50 proteins with the highest degree values and Interaction network form STRING was visualized in R package “visNetwork”. In this study, node degree was used as the primary metric to define putative hub proteins.

### Parallel reaction monitoring validation

PRM validation of candidate proteins was performed in the discovery cohort, including 10 MG patients and 10 healthy controls. The workflow included protein extraction, trypsin digestion, LC–MS/MS analysis, targeted peak integration, and quantitative analysis. PRM verification was performed in the same discovery cohort used for DIA screening, whereas ELISA validation was conducted in an independent validation cohort. For PRM analysis, samples were digested with trypsin, and the resulting peptides were dissolved in mobile phase A for separation using an EASY-nLC 1,200 ultrahigh-performance liquid chromatography (UHPLC) system. Mobile phase A consisted of water with 0.1% formic acid and 2% acetonitrile, whereas mobile phase B contained 0.1% formic acid and 90% acetonitrile. The gradient elution conditions were as follows: 0–40 min, 6–20% B; 40–52 min, 20–28% B; 52–56 min, 28–80% B; and 56–60 min, 80% B, with a flow rate of 500 nL/min. After UHPLC separation, the peptides were introduced into the NSI ion source for ionization and analyzed on an Orbitrap Exploris 480 mass spectrometer. The ion source voltage was set to 2,100 V. Both precursor ions and their corresponding fragment ions were detected using high-resolution Orbitrap analysis. The MS1 scan range was set to 414–1,157 m/z with a resolution of 60,000, and MS2 spectra were acquired at a resolution of 15,000. Database searching was conducted using MaxQuant v1.6.15.0, and PRM raw files were processed in Skyline 21.2 for targeted peak integration and quantification. The data normalization was performed using the local normalization method based on local regression embedded in Spectronaut to correct peptide intensity. For PRM assay design, target peptides were selected according to peptide-level evidence in the DIA discovery dataset, uniqueness to the corresponding target protein, absence of missed cleavage, avoidance of labile modification sites where possible, stable chromatographic behavior, and adequate MS response. Each target protein was represented by one or two unique peptides depending on peptide detectability and signal stability. Detailed targeted peptide information, including peptide sequence, precursor m/z, precursor charge, retention-time window, and related PRM parameters, is provided in Supplementary Table S5. Because isotopically labeled synthetic peptides were not used as absolute quantification internal standards in this study, PRM quantification was based on consistently detected peptide peak areas after normalization. Technical replicates were included in PRM measurements where sample availability permitted. Peptide signals were retained only when they showed stable retention times, clear chromatographic peak shapes, co-elution of fragment ions, and reproducible peak areas across technical replicates. Protein-level abundance was calculated from the normalized peak areas of the corresponding target peptides. PRM quality control was based on retention-time stability, peak-shape consistency, fragment-ion co-elution, and peak-area reproducibility. The PRM quality-control data, including peptide retention times and total peak areas across samples/replicates, are provided in Supplementary Table S5. From the 220 proteins meeting the exploratory nominal differential-expression criteria identified by DIA in this cohort, 20 candidates were selected for PRM verification. The PRM panel was not derived from machine-learning feature selection; instead, two complementary strategies were applied for prioritization: Meriggioli and Sanders (2009) function-driven selection based on GO/KEGG/GSEA annotations with emphasis on MG-relevant themes, such as complement/coagulation cascades and actin cytoskeleton/NMJ-structure-related processes, and (Querol and Illa, 2013) network-driven selection of candidate proteins defined by node degree/connectivity in the PPI network. The intersection of these two sets yielded the final 20 candidate proteins, with additional consideration of PRM feasibility, including peptide-level evidence in the discovery DIA data, availability of proteotypic peptides, and stable chromatographic/MS responses.

### Enzyme-linked immunosorbent assay

Serum samples from the independent validation cohort (20 MG patients and 20 age- and sex-matched healthy controls) were used for ELISA quantification. Relative protein levels were measured using commercial ELISA kits from BYabscience (Nanjing, China) to determine the concentrations of PROC (catalog no. BY--EH110491), PROS1 (catalog no. BY--EH1101053), C4BPA (catalog no. BY--EH113700), PFN1 (catalog no. BY--EH112631), and TLN1 (catalog no. BY--EH113325). All procedures were carried out in strict accordance with the manufacturers’ protocols.

### Statistical analysis

All the statistical analyses were conducted via SPSS Statistics version 25.0 and GraphPad Prism version 8.0. Descriptive statistics were used to summarize the data. The Shapiro–Wilk test was applied to assess the normality of continuous variables. Normally distributed data are presented as the mean ± standard deviation (SD), whereas nonnormally distributed data are reported as the median and interquartile range (IQR; Q1–Q3). Categorical variables are expressed as counts and percentages (n, %). Group comparisons were conducted via appropriate statistical tests on the basis of the data type and distribution. For continuous variables, the independent samples t test was used for normally distributed data, whereas the Mann–Whitney U test was employed for nonnormally distributed data. Categorical variables were analyzed via Pearson’s chi-square test or Fisher’s exact test, as appropriate. The diagnostic performance of the candidate biomarkers was evaluated via receiver operating characteristic (ROC) curve analysis, and the area under the curve (AUC) was reported. To evaluate the combined diagnostic performance of the five-protein panel, a binary logistic regression model was constructed using C4BPA, PROC, PROS1, PFN1, and TLN1 as predictors and disease status (MG vs. HC) as the dependent variable. The predicted probability generated by the logistic regression model was used for ROC curve analysis. The AUC, sensitivity and specificity. Given the limited sample size of the PRM cohort, leave-one-out cross-validation (LOOCV) was additionally performed as an exploratory internal validation. For differential protein screening and analyses involving multiple protein comparisons, raw *p* values were adjusted using the Benjamini–Hochberg procedure to control the FDR. FDR-adjusted *p* values are reported as *q* values. A two-sided raw *p* value < 0.05 together with fold change > 1.5 or < 1/1.5 was used as the exploratory nominal screening criterion, whereas an FDR-adjusted *q* value < 0.05 was used to identify proteins that remained statistically significant after multiple-testing correction.

## Results

### Baseline data situation

As shown in Tables 1, 2, there were no statistically significant differences in age or sex between the MG and HC groups (*p* > 0.05), indicating general comparability between the two cohorts. AChR antibody levels were significantly higher in the MG group than in the HC group (*p* < 0.05). MGFA clinical classification was recorded for MG patients and is summarized in the baseline table. However, because of the small number of patients in each MGFA subgroup, no stratified proteomic analysis or severity-protein correlation analysis was performed.

**Table 1:** Clinical characteristics of participants included in the proteomic discovery cohort.

Variable		MG(*n* = 10)	HC(*n* = 10)	F/χ^2^	*p* value
Age		50.60 ± 11.03	50.60 ± 11.14	*F* = 0.004	1.000>0.05
Gender	Female	3(30%)	3(30%)	χ^2^ = 0.000	1.000>0.05
	Male	7(70%)	3(70%)		
AChR antibody(pmol/L)		501.71 ± 74.01**	330.94 ± 53.98	*F* = 0.693	0.000<0.05
MGFA Classification	Class I	1(10%)			
	Class II	5(50%)			
	Class III	4(40%)			
	Class IV	0(0%)			
Clinical Rating Scale	QMG	12.80 ± 5.63			
	MG-ADL	6.30 ± 3.77			

QMG, Quantitative Myasthenia Gravis Score; MG-ADL, Myasthenia Gravis Activities of Daily Living Scale. Autoantibody status: MG cases are AChR+ (*n* = 10, 100%), HCs are negative. Compared with HC, *p < 0.05; **p < 0.01.

**Table 2:** Clinical characteristics of participants included in the validation cohort.

Variable		MG(*n* = 20)	HC(*n* = 20)	F/χ^2^/Z	*p* value
Age		60.40 ± 3.39	60.05 ± 3.23	*F* = 0.047	0.941>0.05
Gender	Female	11(55%)	11(55%)	χ^2^ = 0.000	1.000>0.05
	Male	9(45%)	9(45%)		
AChR antibody(pmol/L)		450.70 ± 17.51**	324.84 ± 18.34	Z = −4.302	0.000<0.05
MGFA Classification	Class I	8(40%)			
	Class II	9(45%)			
	Class III	2(10%)			
	Class IV	1(5%)			
Clinical Rating Scale	QMG	15.35 ± 5.58			
	MG-ADL	6.35 ± 2.74			

Autoantibody status: MG cases are AChR+ (*n* = 20, 100%), HCs are negative. Compared with HC, *p < 0.05; **p < 0.01.

### Protein identification results

**Figure 1:**
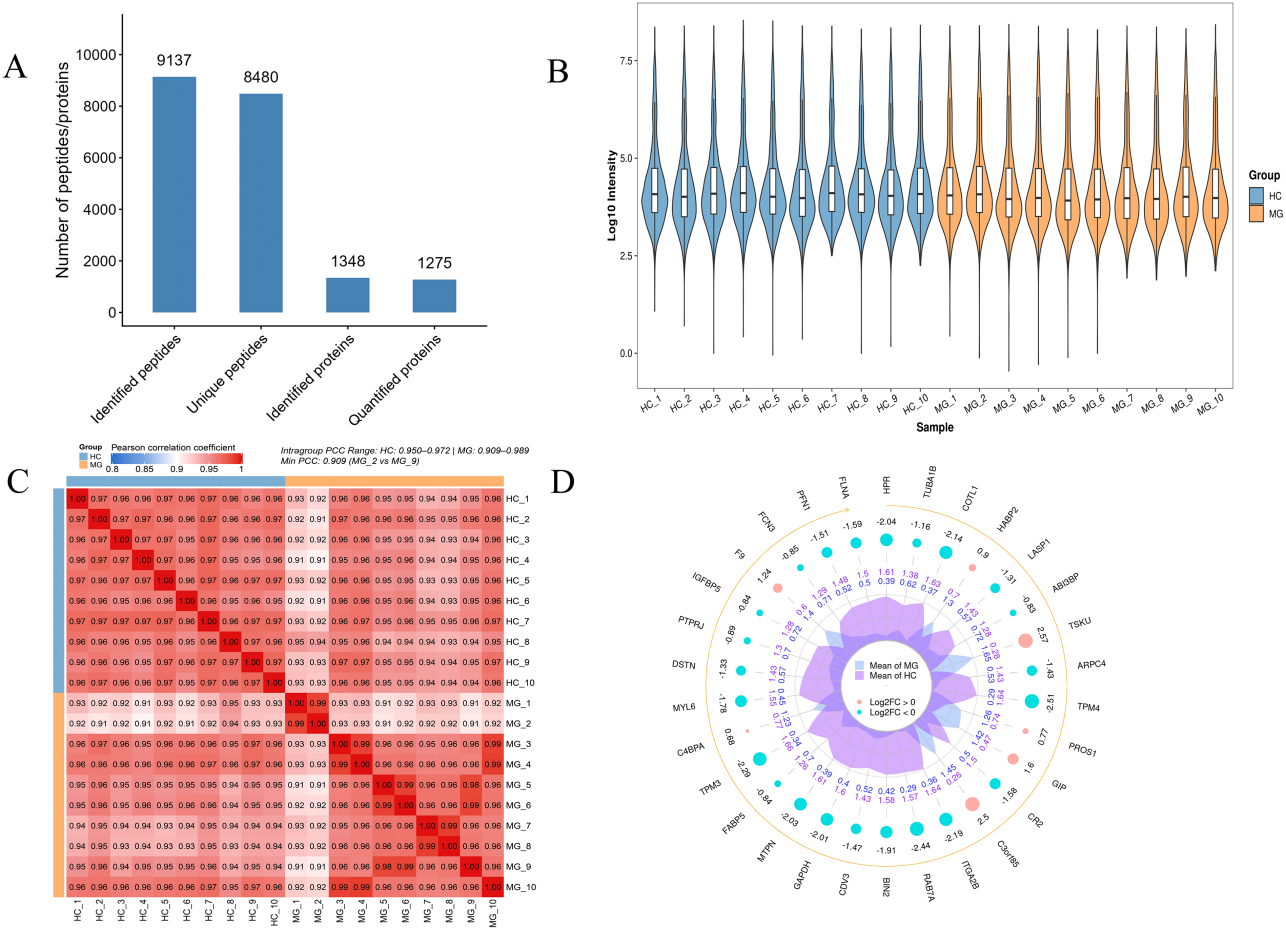
Overview and quality-control assessment of DIA-based serum proteomic analysis. **(A)** Summary of identified peptides, unique peptides, detected proteins, and quantitatively comparable proteins. **(B)** Distribution and variability of log10-transformed protein intensity values across samples shown by combined violin and box plots. Zero intensity entries were masked as missing values before log10 transformation. The x-axis indicates sample identifiers, the y-axis indicates log10-transformed protein intensity values, and colors indicate MG and HC groups. The median log10 intensity values across all 20 biological samples ranged from 3.915 to 4.105. **(C)** Sample correlation analysis and reproducibility assessment. Pairwise Pearson correlation coefficients were calculated using log10-transformed protein intensity values with pairwise complete observations. The within-group PCC ranged from 0.950 to 0.972 in the HC group and from 0.909 to 0.989 in the MG group. **(D)** Radar plot of the top 30 differentially expressed proteins ordered clockwise by ascending *p* value. Pink and light blue indicate upregulated and downregulated proteins, respectively, and dot size represents the magnitude of log2-transformed fold change. The inner layer shows the average quantitative abundance in the MG and HC groups.

Mass spectrometry analysis was performed to profile the proteomes of the HC and MG groups. A total of 9,137 peptides were identified, of which 8,480 were unique peptides. In total, 1,348 proteins were detected, among which 1,275 were quantitatively comparable between the two groups (Figure 1A).

### Distribution and variability of protein intensities across samples

To assess the distribution and variability of protein intensities across samples, log10-transformed protein intensity values were extracted and visualized using combined violin and box plots (Figure 1B). The x-axis represents individual sample identifiers, the y-axis shows log10-transformed intensity values, and the fill colors denote sample groupings. Zero intensity entries were masked as missing values before log10 transformation. To quantitatively support data consistency, the median log10-transformed protein intensity values across all 20 biological samples were calculated and ranged from 3.915 to 4.105. The comparable interquartile ranges and broadly similar intensity density profiles across samples indicated consistent global protein intensity distributions, supporting the suitability of the dataset for subsequent comparative analyses.

### Reproducibility analysis of sample data

To evaluate sample reproducibility, pairwise PCCs were calculated across all samples based on log10-transformed protein intensity values, and a correlation heatmap was generated for visualization (Figure 1C). Missing values were handled using pairwise complete observations. The Pearson coefficient quantifies the degree of linear correlation between two samples, with values closer to 1 indicating stronger similarity. The within-group PCC ranged from 0.950 to 0.972 in the HC group and from 0.909 to 0.989 in the MG group. These quantitative PCC ranges support good within-group consistency and improve the reproducibility assessment beyond visual inspection of the heatmap.

### Identification of differentially expressed proteins

DEPs were initially identified using the exploratory nominal criteria of fold change > 1.5 or < 1/1.5 and raw *p* < 0.05. Based on these criteria, 220 proteins showed differential abundance between MG patients and HCs, including 49 upregulated and 171 downregulated proteins. After Benjamini–Hochberg FDR correction, 138 proteins remained statistically significant, including 23 upregulated and 115 downregulated proteins. Notably, the five prioritized proteins selected for subsequent PRM and ELISA validation—PROS1, PROC, C4BPA, PFN1, and TLN1—were all retained among the FDR-significant proteins. The complete differential analysis results, including raw *p* values and FDR-adjusted *q* values, are provided in Supplementary Table S1. Downstream exploratory enrichment analyses were based on the 220 proteins meeting the nominal differential-expression criteria. A radar plot was generated to visualize the relative expression levels of the top 30 DEPs (Figure 1D).

### GO, KEGG, and GSEA functional enrichment analyses

**Figure 2:**
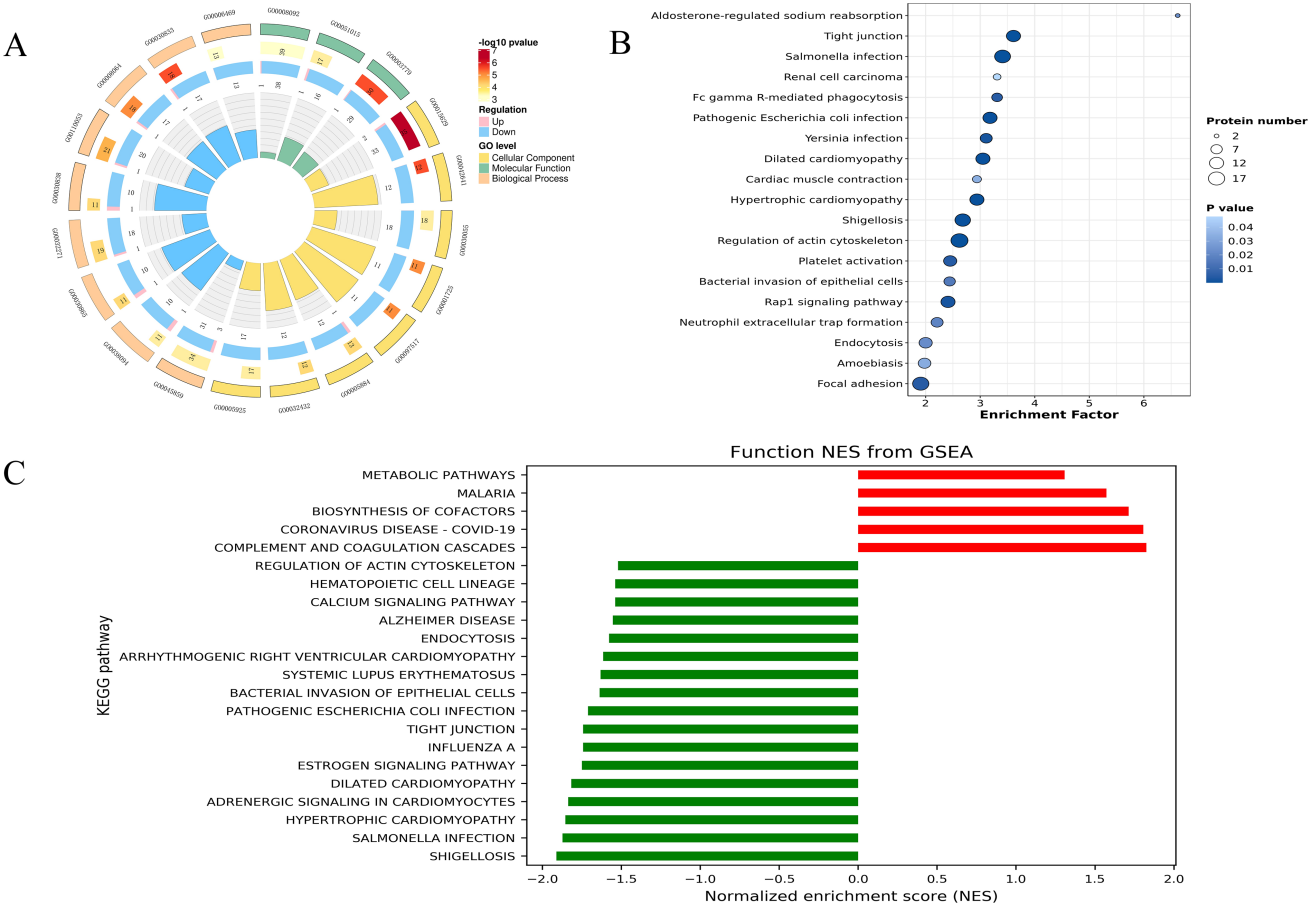
Functional enrichment analyses of DEPs between the MG and HC groups. **(A)** GO enrichment analysis of DEPs between the MG and HC groups. All selected pathways were significantly enriched. Circos track definitions (outer to inner): Track 1-enriched GO terms colored by category. Track 2-number of differential proteins per term and –log10(*p*-value); redder color indicates greater significance. Track 3-bar chart of upregulated (pink) and downregulated (blue) differential protein counts. Track 4-fold enrichment. Terms with <4 entries are omitted; **(B)** KEGG pathway enrichment analysis of DEPs between the MG and HC groups. The y-axis lists enriched KEGG pathways, the x-axis represents fold enrichment or enrichment ratio, bubble size indicates the number of proteins involved in each pathway, and color indicates the corresponding *p* value; **(C)** GSEA of DEPs between the MG and HC groups. In GSEA, all proteins are first ranked by their ratio values (MG/HC comparison). GSEA tests whether a functional set is enriched at the top or bottom of this ranked list. Top enrichment indicates an overall positive trend for that set, and bottom enrichment indicates a negtive trend. Enrichment results for positive and negative function are combined and filtered at *p* < 0.05 to generate a bar chart. The x-axis shows NES, and the y-axis shows functional sets. Red: positive sets (NES > 0); green: positive sets (NES < 0).

To characterize the biological themes associated with the DEPs, GO enrichment analysis was performed and visualized using a multiring enrichment plot (Figure 2A). At the cellular component level, enriched terms included actin cytoskeleton (GO:0015629), stress fibers (GO:0001725), and focal adhesions (GO:0005925), indicating that a subset of DEPs was annotated to cytoskeleton- and adhesion-related cellular structures. ACTB and TLN1 were representative proteins mapped to these categories. At the molecular function level, enrichment of actin binding (GO:0003779) was observed, with actin-dynamics-related proteins such as TLN1 and PFN1 contributing to this annotation. At the biological process level, terms related to actin filament polymerization (GO:0030833) and Fcγ receptor signaling pathway (GO:0038094) were enriched, suggesting pathway-level associations between immune-related signaling and cytoskeleton-associated processes in MG serum proteomic profiles. On the basis of the GO findings, KEGG pathway enrichment analysis was conducted to further explore the functional distribution of the DEPs (Figure 2B). Compared with HCs, DEPs in MG patients were mainly enriched in tight junctions, regulation of the actin cytoskeleton, and the Rap1 signaling pathway. These pathways are related to cell adhesion, cytoskeletal organization, and signal transduction, but the enrichment results should be interpreted as pathway-level associations rather than direct evidence of structural damage at the neuromuscular junction. GSEA was further employed to evaluate whether predefined gene sets showed coordinated enrichment trends between MG patients and HCs (Figure 2C). The GSEA results indicated that proteins relatively increased in MG were enriched in complement and coagulation cascades, whereas proteins relatively decreased in MG were enriched in tight junctions and regulation of the actin cytoskeleton. These findings were broadly consistent with the GO and KEGG enrichment results and suggest that complement/coagulation regulation and cytoskeleton/adhesion-related processes may represent prominent pathway-level signals in this exploratory proteomic dataset. However, enrichment analyses alone do not establish causal pathway activation or a direct “coagulation-complement-inflammation-cytoskeleton damage cascade”; further functional experiments are required to validate these mechanistic links.

### Cluster analysis results

**Figure 3:**
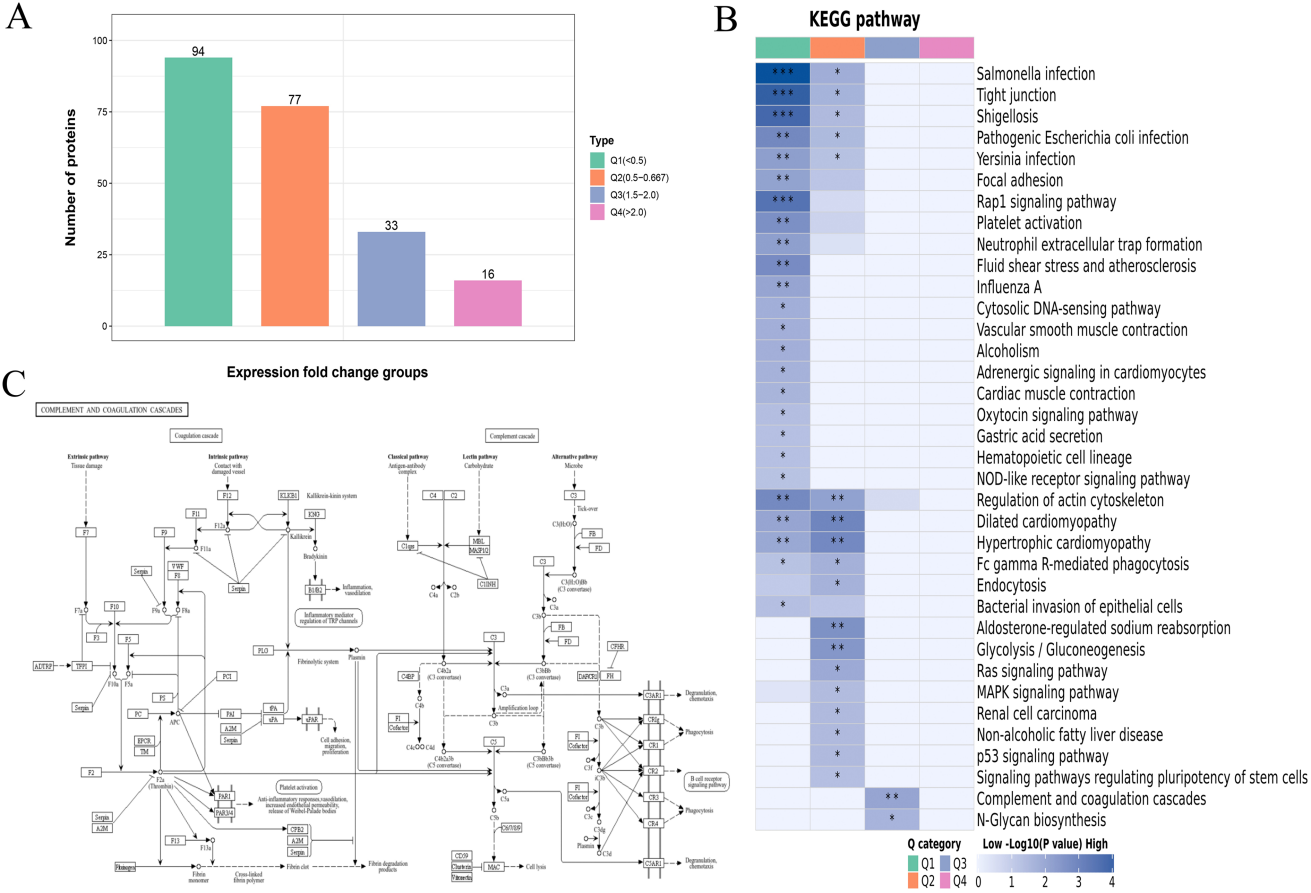
Functional clustering of DEPs. **(A)** Groups of proteins according to differential expression fold changes. To compare functional similarities and differences among proteins with different fold changes, the proteins were divided into four groups (Q1 to Q4) using fold change thresholds of 1.5 and 2. Specifically, proteins with a fold change less than 1/2 (<0.5) were assigned to Q1; those with a fold change between 1/2 and 1/1.5[0.667] were assigned to Q2; those with a fold change between 1.5 and 2 were assigned to Q3; and those with a fold change greater than 2 were assigned to Q4. Proteins with fold changes in the range of 0.667–1.5 (no significant differential expression) were not included in this analysis; **(B)** Hierarchical clustering heatmap of KEGG pathway enrichment across quartiles. Based on the Fisher’s exact test *p*-values obtained from enrichment analysis, hierarchical clustering was performed to group related functions across different Q-groups, and a heatmap was generated. The horizontal axis of the heatmap represents different Q-groups, and the vertical axis represents KEGG pathway functions enriched by differentially expressed proteins. The color of each cell corresponds to the significance level of enrichment for a given Q-group and function. Blue indicates high enrichment significance, while blue-white indicates low enrichment significance. Asterisks denote significance levels: ^*^ for *p* < 0.05, ^**^ for *p* < 0.01, and ^***^ for *p* < 0.001; **(C)** KEGG pathway map of the complement and coagulation cascades.

To compare the functional similarities and differences among proteins with varying fold changes, the differentially expressed proteins were categorized into four quartiles (Q1–Q4) on the basis of their fold change values (Figure 3A). KEGG enrichment analysis was then performed for each quartile. The resulting Fisher’s exact test *p* values were subjected to hierarchical clustering, enabling the grouping of related functional categories across the quartiles, which were visualized as a heatmap (Figure 3B). Notably, Q3 (fold change of 1.5–2.0) exhibited the most significant association with the complement and coagulation cascades pathway (hsa04610). The differentially expressed proteins within this pathway are listed in Figure 3C. Further mechanistic studies are warranted to elucidate the specific biological roles of these proteins. Although multiple pathways were enriched, the complement and coagulation cascade emerged as one of the most prominent pathway-level signals in this exploratory proteomic dataset.

### PPI network construction

**Figure 4:**
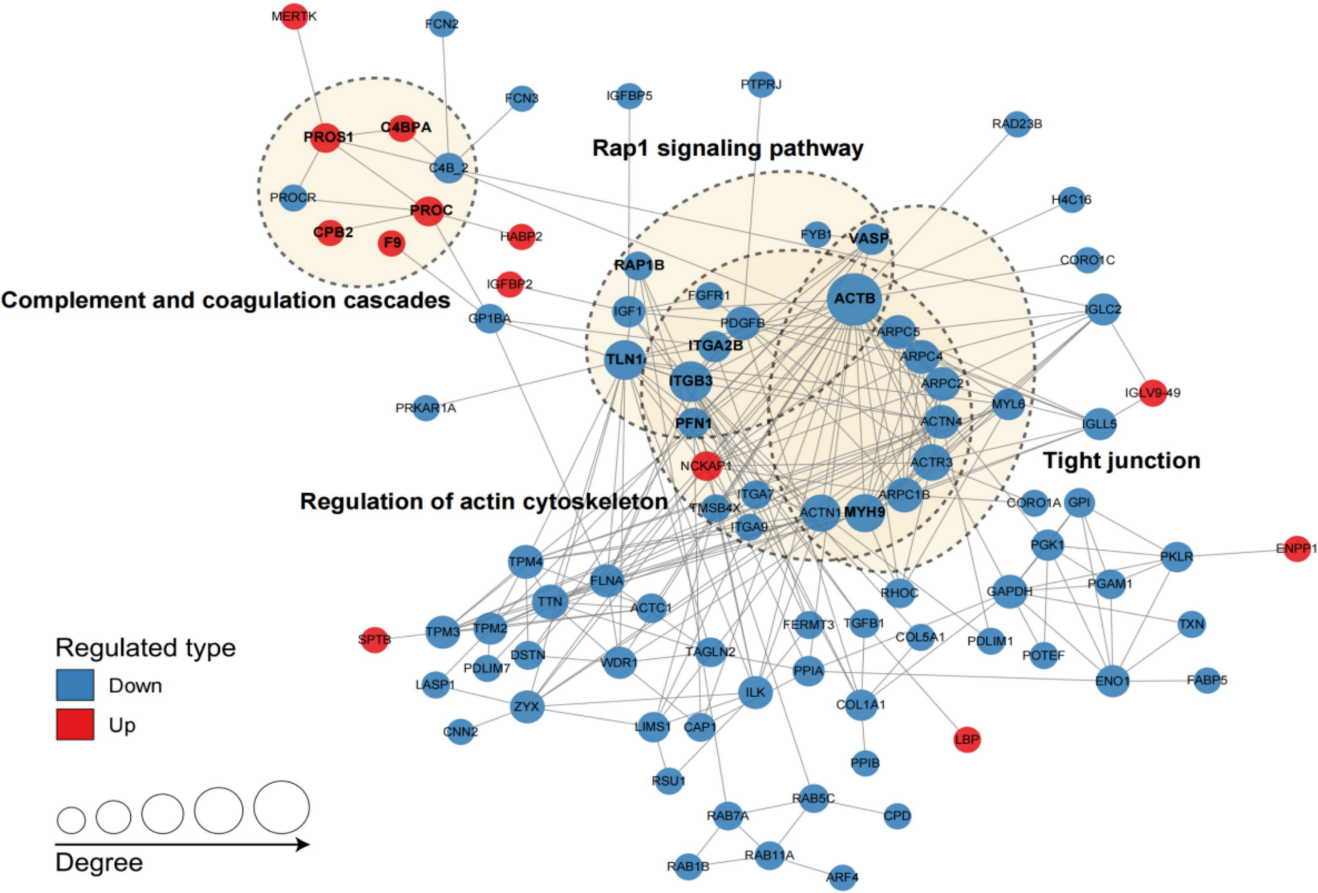
PPI network analysis. The PPI network was constructed using STRING with a combined confidence score ≥0.7. Nodes represent proteins, and edges represent predicted or known protein–protein interactions. Node size indicates degree centrality, edge thickness indicates the STRING combined interaction score, and node color indicates clustering modules identified by the Markov clustering algorithm. The top 50 high-connectivity proteins were visualized.

To further explore the molecular relationships among the DEPs, we constructed a high-confidence PPI network using STRING (combined confidence score ≥ 0.7). After filtering, the network comprised 131 nodes and 280 edges, with a mean node degree of 4.27. The full interaction table was used to calculate node degree, after which the 50 most highly connected proteins were extracted to generate a high-connectivity subnetwork, which is shown in Figure 4. Tightly connected modules were delineated using STRING’s default Markov clustering algorithm (MCL), and the network was visualized in visNetwork with the STRING combined score used as the edge weight. This subnetwork highlights several biological themes that were also enriched in GO and KEGG analyses. Complement and coagulation-related proteins (e.g., PROS1, PROC, C4BPA) formed a tightly linked cluster, consistent with the known involvement of complement activation in MG. Cytoskeleton-associated proteins (e.g., PFN1, MYH9, TLN1, VASP) appeared as high-degree nodes and mapped to pathways such as actin cytoskeleton regulation and Rap1 signaling, with ACTB representing one of the highest-connectivity nodes in the subnetwork. In this network, proteins with higher degree values were considered hub nodes. These observations suggest that DEPs with high network connectivity converge on biological processes relevant to MG pathophysiology, including complement/coagulation cascades and cytoskeletal/adhesion dynamics. However, these findings should be interpreted as functional network associations rather than biomarker evidence.

### PRM validation

**Figure 5:**
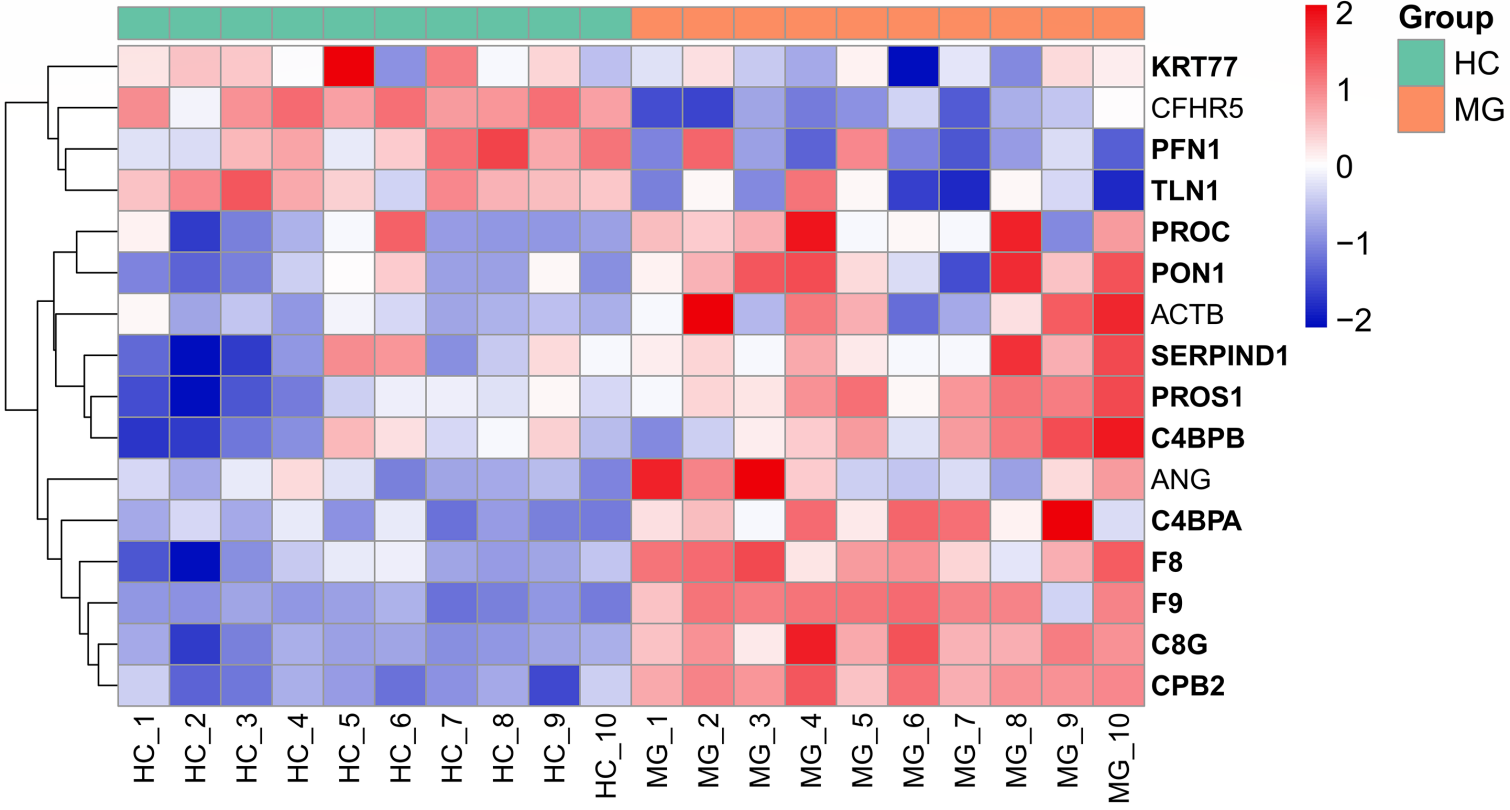
PRM validation heatmap of prioritized candidate proteins. Columns represent individual serum samples, and rows represent candidate proteins quantified by PRM. Sample groupings are indicated as MG or HC above the heatmap. Red indicates relatively higher normalized protein abundance, whereas blue indicates relatively lower abundance. Protein names are shown on the y-axis, and proteins with expression trends consistent with DIA proteomics are highlighted in bold.

**Figure 6:**
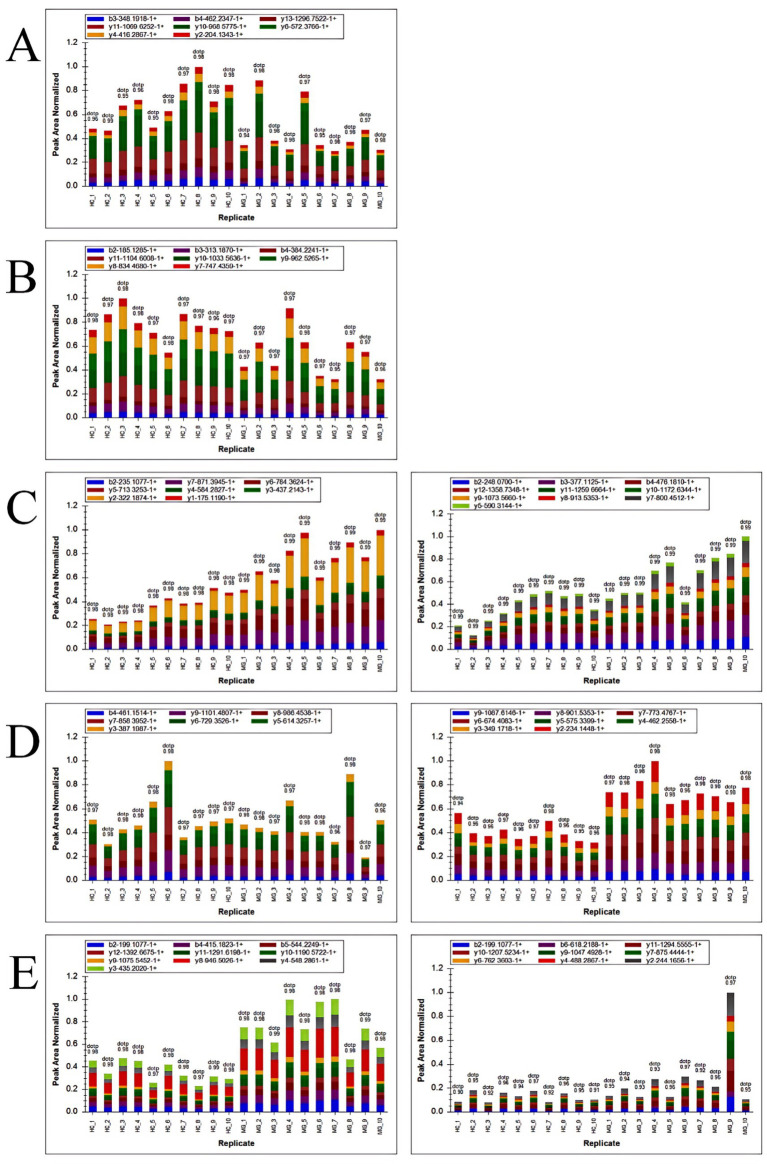
Panels **A–E** represent the fragment ion peak area distributions of PFN1 (P07737), TLN1 (Q9Y490), PROS1 (P07225), PROC (P04070), and C4BPA (P04003), respectively. The x-axis indicates peptide fragments or sample groups, and the y-axis indicates integrated peak area. These plots were used to evaluate PRM signal consistency and peptide-level quantification.

PRM quantification was performed on the basis of peak area measurements. In the experimental design, two unique peptides were targeted for each protein whenever possible; however, for a subset of targets, only one peptide could be reliably detected due to sensitivity or other analytical constraints. We selected 20 candidate proteins for PRM validation using a clinically anchored prioritization strategy rather than machine-learning feature selection. Specifically, candidates were shortlisted from the differential proteins by integrating enrichment results (GO/KEGG/GSEA) and PPI analysis, prioritizing proteins with stronger relevance to MG pathophysiology (complement/coagulation and actin cytoskeleton/adhesion processes), larger fold changes, more peptide support and more stable PRM quantification, and prior implication in published MG studies; information on highly connected (high-degree) core nodes from the PPI network was also considered to strengthen biological plausibility. Practical PRM feasibility (peptide evidence and detectability) was also considered. As shown in the heatmap (Figure 5), while 16 of the 20 candidates were successfully quantified and 13 exhibited consistent expression patterns with the proteomics results, discrepancies in expression trends were observed for certain proteins; ANG, ACTB, and CFHR5 showed discordant trends relative to the DIA results. These discrepancies may partly reflect batch effects in the DIA data, which can cause varying trends between different sample batches, and single-peptide quantification may be more susceptible to experimental variability. Based on these findings, PFN1 (MG/HC = 0.653, *p* = 0.0055) and TLN1 (MG/HC = 0.672, *p* = 0.0030) were significantly downregulated in MG patients compared with HCs, whereas PROS1 (MG/HC = 1.988, *p* = 1.02 × 10^−4^), PROC (MG/HC = 1.293, *p* = 0.0118), and C4BPA (MG/HC = 2.152, *p* = 1.51 × 10^−4^) were markedly upregulated. The fragment ion peak area distributions for peptides corresponding to these five proteins are presented in Figure 6.

### ROC curve analysis of DEPs

**Figure 7:**
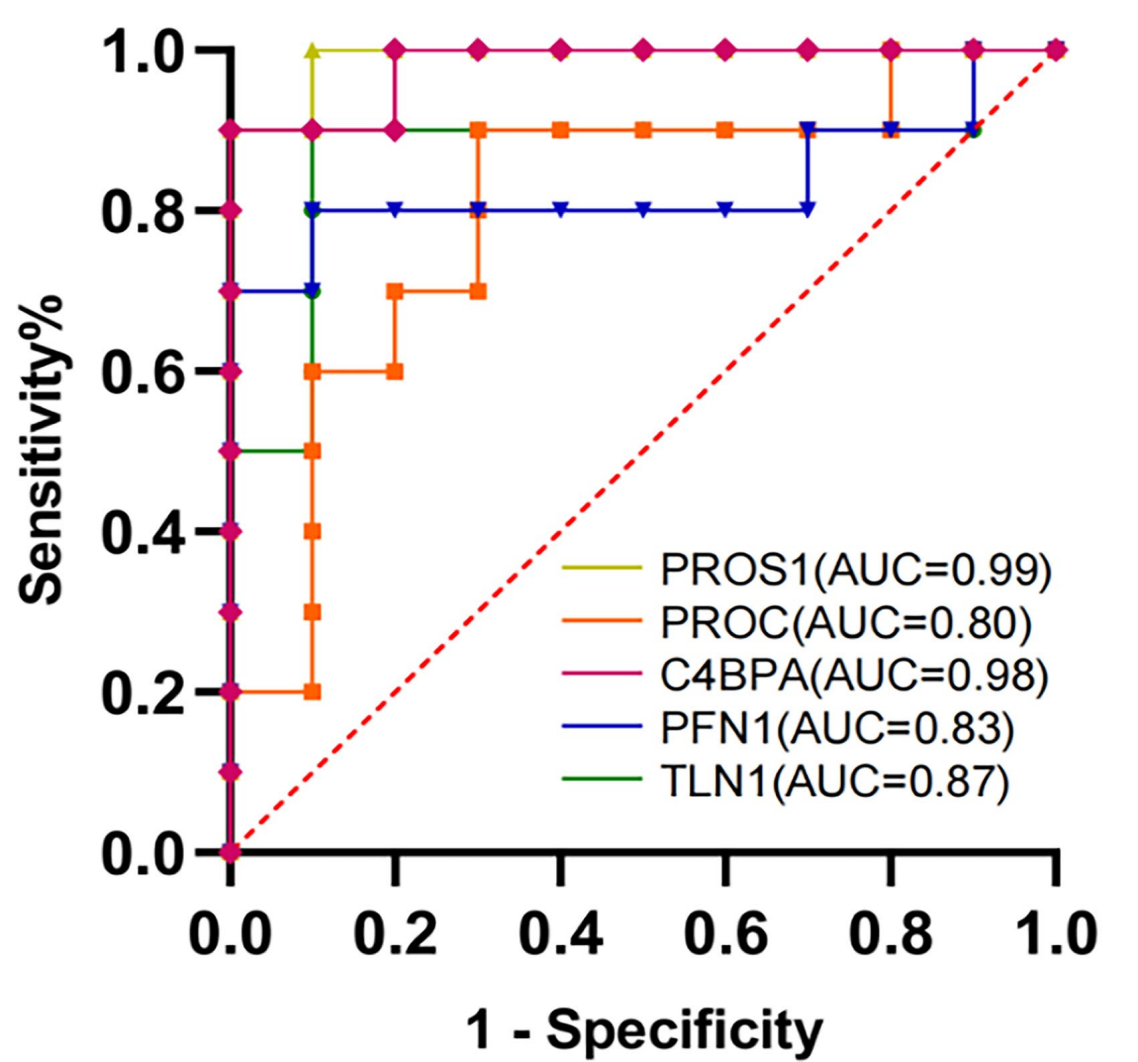
ROC curves of PROS1, PROC, C4BPA, PFN1, and TLN1. The x-axis represents the false-positive rate, and the y-axis represents the true-positive rate. AUC values and 95% confidence intervals are shown for each candidate protein. Because of the limited cohort size, these ROC results should be interpreted as preliminary.

ROC curve analysis was performed to evaluate the diagnostic performance of the identified biomarkers based on the PRM validation dataset, which served as the discovery-phase verification cohort in this study. In the ROC plots, the x-axis represents the false positive rate and the y-axis represents the true positive rate. The discriminative ability of each biomarker was quantified by the area under the curve (AUC), with 0.5 indicating random performance and values approaching 1 indicating strong diagnostic discrimination. As shown in Figure 7, the AUC values for PROS1, PROC, C4BPA, PFN1, and TLN1 were 0.99, 0.80, 0.98, 0.83, and 0.87, respectively. Corresponding 95% confidence intervals (CIs) were 0.9583–1.000 (PROS1), 0.5948–1.000 (PROC), 0.9295–1.000 (C4BPA), 0.6230–1.000 (PFN1), and 0.6871–1.000 (TLN1). Based on the AUC and CI results, the overall sensitivity ranking of the biomarkers was: PROS1 > C4BPA > TLN1 > PFN1 > PROC. To further evaluate the combined diagnostic value of the five prioritized proteins, a binary logistic regression model was established using C4BPA, PROC, PROS1, PFN1, and TLN1 as predictors. The predicted probability from this model was used to generate a combined ROC curve. In the PRM cohort, the five-protein panel achieved an apparent AUC of 1.000, with a sensitivity of 100.0% and a specificity of 100.0% at the optimal cutoff determined by the Youden index. To reduce the risk of overestimating diagnostic performance, leave-one-out cross-validation was additionally performed. The LOOCV-based ROC analysis yielded an AUC of 0.950, with a sensitivity of 90.0% and a specificity of 90.0%. Given the small sample size and the lack of internal cross-validation, these AUC estimates should be considered preliminary and may overestimate diagnostic performance. Therefore, they require confirmation in larger independent cohorts.

### ELISA-based validation of candidate biomarkers

**Figure 8:**
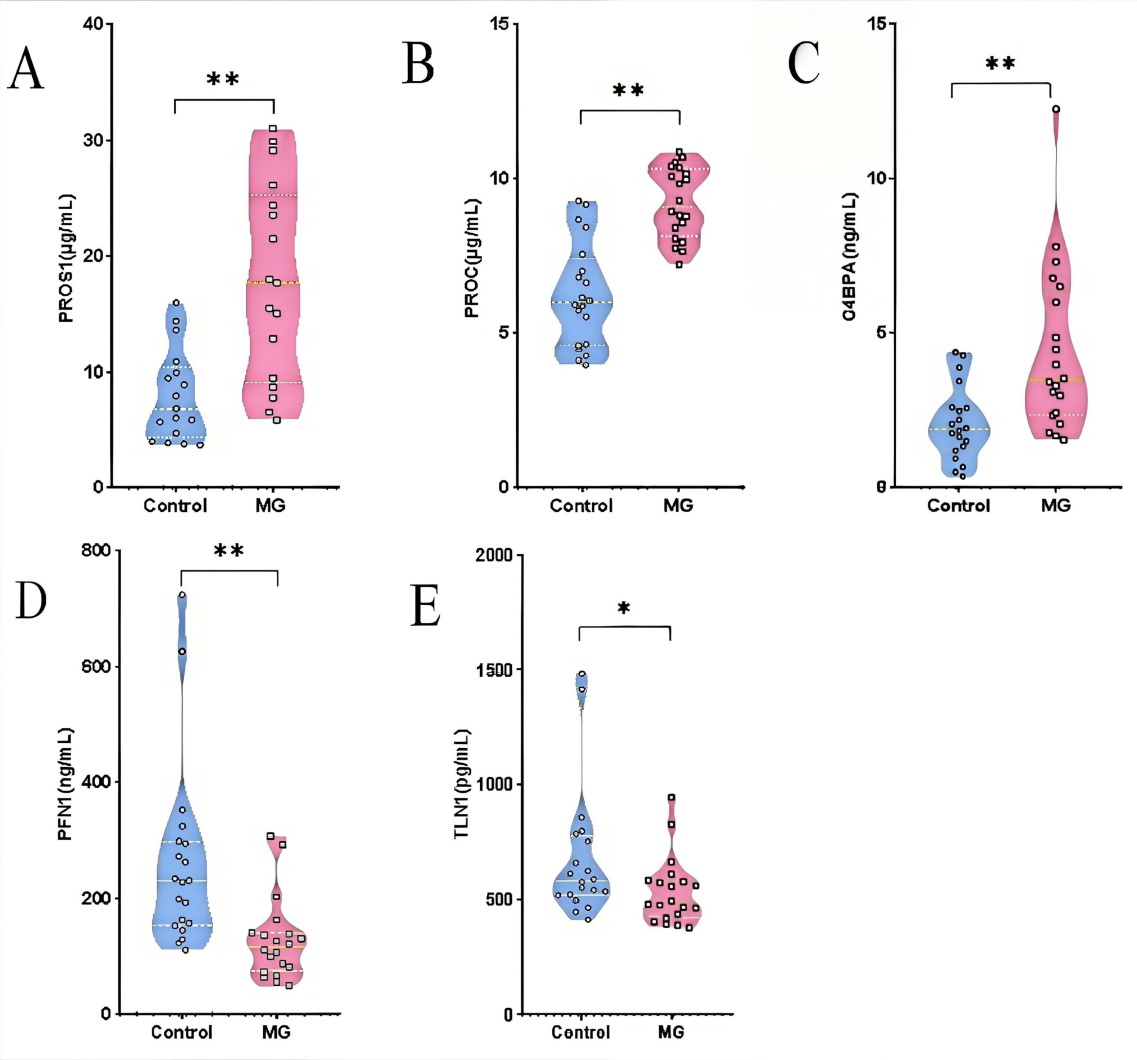
Validation of candidate biomarkers in myasthenia gravis (MG) and healthy controls (HC) by ELISA. Violin plots compare serum levels between HC and MG groups. **(A)** PROS1, **(B)** PROC, **(C)** C4BPA, **(D)** PFN1, and **(E)** TLN1. Each dot represents an individual sample; violin plots illustrate data distribution and median values. MG patients showed significantly higher levels of PROS1, PROC, and C4BPA, and significantly lower levels of PFN1 and TLN1 compared with HC. Statistical significance is indicated as *p < 0.05; **p < 0.01.

The above analyses suggested that PROS1, PROC, C4BPA, PFN1, and TLN1 may serve as potential protein biomarkers for the diagnosis of MG. To validate these findings, ELISA was performed to quantify the serum levels of these proteins, as shown in Figure 8. Compared with those in the HCs, the serum levels of PROS1, PROC, and C4BPA were significantly elevated in the MG group (*p* < 0.01), whereas the PFN1 and TLN1 levels were significantly lower (*p* < 0.05 and *p* < 0.01, respectively). These results provide preliminary validation of the differential abundance of PROS1, PROC, C4BPA, PFN1, and TLN1 in AChR-positive MG. However, their diagnostic utility and disease specificity require further validation in larger cohorts and disease-control populations.

## Discussion

MG is an autoimmune disorder of NMJ that is mediated by pathogenic autoantibodies. Its diagnosis relies primarily on clinical manifestations, detection of AChR antibodies, and neuroelectrophysiological testing (Conti-Fine et al., 2006). However, several challenges persist in clinical practice: in approximately 15% of patients with generalized MG and 50% of patients with ocular MG, both AChR and MuSK antibodies are absent, complicating diagnosis (Vinciguerra et al., 2023); currently available biomarkers have limited predictive value for disease activity and therapeutic responsiveness (Kaminski et al., 2012); the clinical manifestations of MG can mimic those of various neurological and systemic disorders, contributing to a considerable rate of misdiagnosis (Meriggioli and Sanders, 2009; Andrapalliyal et al., 2023); and diagnostic delay in MG is further exacerbated by younger age at onset, subtle or fluctuating early symptoms, and prolonged intervals before antibody confirmation (Marlet et al., 2024). These challenges underscore the urgent need to identify novel biomarkers for early diagnosis and precision intervention. These unmet needs underscore the urgency for novel biomarkers to enable earlier diagnosis and guide precision therapy.

To address this gap, we performed serum proteomic profiling in a discovery cohort of 10 AChR-positive MG patients and 10 HCs, followed by PRM verification in the same discovery cohort and ELISA validation in an independent cohort of 20 MG patients and 20 HCs. This revealed 220 proteins meeting the exploratory nominal differential-expression criteria, of which 138 remained significant after BH-FDR correction, with pathway enrichment highlighting the complement and coagulation cascades and actin cytoskeleton regulation as prominent pathway-level alterations. Using this discovery–PRM verification–ELISA validation strategy, we report that a panel of five proteins—PROS1, PROC, C4BPA, PFN1, and TLN1—may represent an exploratory candidate serum biomarker panel for AChR-positive MG. We initially employed serum proteomics to compare protein profiles between HCs and MG patients comprehensively. Functional enrichment analysis revealed four biological pathways closely associated with MG: complement and coagulation cascades, actin cytoskeleton regulation, Rap1 signaling, and tight junctions. PPI network analysis further identified high-connectivity candidate proteins, including PROC, PROS1, C4BPA, F9, and CPB2 in the complement/coagulation cascades; ACTB, ITGA2B, ITGB3, PFN1, and MYH9 among 18 proteins involved in actin cytoskeleton regulation; RAP1B, TLN1, ITGA2B, ITGB3, PFN1, VASP, and ACTB in the Rap1 pathway; and VASP, ACTB, and MYH9 in tight junctions. Targeted PRM verification supported the altered abundance of 16 out of 20 candidate proteins in the discovery cohort. Based on their centrality in the PPI network and functional convergence across multiple enriched pathways, we prioritized three upregulated proteins (PROS1, PROC, and C4BPA) and two downregulated proteins (PFN1 and TLN1) as candidate proteins for further evaluation. This selection was driven by their centrality within the PPI network and functional convergence across four key pathways: PROC, PROS1, and C4BPA, which are major regulators of complement/coagulation, and PFN1 and TLN1, which are key modulators of actin cytoskeletal dynamics and cell adhesion. ROC curve analyses, coupled with ELISA measurements in the independent cohort, provided preliminary support for their discriminatory potential. However, the proposed coagulation–complement–inflammation–cytoskeleton framework should be interpreted as a pathway-level, hypothesis-generating model derived from enrichment and network analyses rather than as an experimentally demonstrated causal cascade.

PROS1 (protein S) is a vitamin K–dependent glycoprotein synthesized by hepatic endothelial cells and megakaryocytes. As a ligand of TAM receptor tyrosine kinases (Tyro3/Axl/Mer), PROS1 modulates inflammation and efferocytosis through activation of the Gas6–PROS1 pathway (Lemke, 2013; Stitt et al., 1995). PROC (protein C) is a serine protease zymogen whose activated form (APC) inhibits coagulation by degrading factors Va and VIIIa and exerts cytoprotective and anti-inflammatory effects (Sarangi et al., 2010). In MG, chronic autoimmune activation may underlie the observed upregulation of both PROS1 and PROC. Since excessive complement activation in MG leads to NMJ postsynaptic membrane injury, PROS1 and PROC may attenuate damage by limiting C3b/C5b-9 deposition, thereby reducing MAC formation. However, their compensatory increase appears insufficient to fully counteract antibody-mediated complement activation, suggesting their potential as therapeutic targets.

C4BPA (C4b-binding protein *α*-chain) is a pivotal regulator of the classical complement pathway and inhibits C3 convertase assembly by binding C4b and promoting factor I–mediated degradation (Blom et al., 2003). In MG—especially in individuals positive for anti-AChR antibodies—activation of the classical pathway by these autoantibodies drives destruction of the NMJ via MAC formation (Ozawa et al., 2023). The observed increase in C4BPA likely reflects a compensatory response aimed at restraining excessive complement activity and preserving neuromuscular integrity. Interestingly, analogous complement regulators, such as CD55 (decay-accelerating factor, DAF) and CD59, have been shown to moderate complement activation and limit tissue injury in MG models (Kaminski et al., 2006). Moreover, increases in soluble complement regulatory proteins—including vitronectin and clusterin—have been reported in MG patient sera (Ozawa et al., 2021), further indicating a systemic attempt to counterbalance complement overactivation. Taken together, the inverse correlation between C4BPA levels and clinical severity supports its potential as a biomarker of disease activity and a potential guide for complement-targeted therapies.

Profilin-1 (PFN1) serves as a central coordinator of actin monomer availability, poly-L-proline (PLP) motif binding and phosphoinositide-mediated signaling, thereby safeguarding cytoskeletal stability and adaptive remodeling. Mutations that impair these interactions—such as those affecting the actin- or PLP-binding domains—disrupt filament turnover, alter intracellular trafficking, and can trigger protein misfolding or aggregation (Alkam et al., 2017). In neuromuscular contexts, such perturbations may compromise synaptic architecture and axonal transport, increasing the susceptibility of NMJs to immune-mediated injury. Given that MG pathogenesis involves both antibody-driven postsynaptic damage and inflammatory remodeling of muscle tissue, it is plausible that chronic neuromuscular stress, proteolytic degradation, or reduced muscular synthesis could contribute to the observed decline in circulating PFN1. This perspective positions PFN1 not only as a structural regulator but also as a potential downstream marker of immune-related cytoskeletal disruption in MG.

TLN1 (Talin-1), a high-molecular–weight cytoskeletal protein, orchestrates integrin–actin cytoskeletal linkage and focal adhesion dynamics, and is critical in mechanotransduction across diverse tissues (Zhao et al., 2022). Given its ubiquitous expression and central role in cell–matrix communication, perturbations in Talin-1 could influence immune cell trafficking or synaptic stability, both of which are compromised in MGs. The observed Talin-1 reduction in MG serum contrasts with prior findings in multiple sclerosis (MS), where elevated serum soluble Talin-1 (sTalin-1) levels were correlated with active disease phases and a worse short-term prognosis (Muto et al., 2017). This dichotomy suggests disease-specific modulation of Talin-1 processing or release, potentially reflecting divergent immune pathomechanisms. In MG, impaired NMJ integrity and thymic abnormalities predominate, suggesting that decreased Talin-1 may reflect early synaptic alterations or disrupted focal adhesion signaling in muscle or immune tissues. Furthermore, oncological studies have revealed that altered Talin-1 expression is correlated with tumor invasiveness and metastatic potential, reinforcing the notion that its dysregulation has pronounced effects on cell adhesion and motility (Rezaie et al., 2023). Although MG is an autoimmune rather than neoplastic condition, analogous principles may apply: Talin-1 downregulation could destabilize the NMJ structure or modify immune cell adhesion dynamics.

Collectively, PROS1, PROC, C4BPA, PFN1, and TLN1 map to a proposed “coagulation–complement–inflammation–cytoskeleton” framework that may be relevant to MG pathophysiology. Dysregulation of coagulation/complement regulators (PROS1, PROC, and C4BPA) may modulate NMJ injury, while persistent autoimmune-driven complement activation can sustain a proinflammatory microenvironment. Because our analyses are based on serum proteomics, these protein alterations are more likely to reflect systemic immune and inflammatory responses in MG rather than direct structural damage at the neuromuscular junction, and should be interpreted accordingly as circulating markers of broader immunopathology. The observed changes in PFN1 and TLN1 are consistent with cytoskeletal pathway involvement in MG and may indicate downstream disturbances in actin dynamics or adhesion processes; however, these findings do not demonstrate a direct causal role in neuromuscular junction remodeling and should be interpreted as hypothesis-generating.

Beyond MG, several proteins in our panel have also been reported in broader autoimmune or neuromuscular diseases. C4BPA encodes the *α*-chain of C4b-binding protein (C4BP), a major regulator of the classical complement pathway; C4BP has been implicated in systemic autoimmune conditions and idiopathic inflammatory myopathies, where it modulates complement activation and local inflammatory damage (Serrano et al., 2022). Protein S (PROS1) and protein C (PROC) are central anticoagulant factors with additional anti-inflammatory and immunoregulatory roles, and reduced PROS1–TAM signaling has been linked to chronic inflammatory and autoimmune states (Carrera Silva et al., 2013). On the neuromuscular side, PFN1 mutations are a well-established genetic cause of familial amyotrophic lateral sclerosis and disrupt actin polymerization and axonal dynamics (Wu et al., 2012), whereas talin-1 (TLN1), which links integrins to the actin cytoskeleton, has been identified as a serum autoantigen and elevated soluble marker in multiple sclerosis, suggesting a role in immune–cytoskeletal crosstalk in demyelinating disease (Muto et al., 2015). These cross-disease observations support the biological plausibility of our panel but also indicate that some components may reflect systemic immune activation shared across immune-mediated disorders rather than MG-specific signatures. These observations also caution against interpreting the present protein panel as MG-specific before validation against other autoimmune and neuromuscular disease controls.

In this exploratory cohort, the five-protein panel showed preliminary discriminatory performance, with AUC values greater than 0.80. However, because all enrolled MG patients were anti-AChR antibody–positive by design and the sample size was limited, the current data do not establish clinical diagnostic utility or applicability to seronegative, MuSK-positive, LRP4-positive, ocular, or atypical MG. These possibilities should be examined in future larger and clinically stratified cohorts.

Several recent proteomic and multi-omics studies have examined circulating biomarkers in MG, and a direct comparison with these studies helps clarify the relative novelty and limitations of our findings. Bhandage et al. identified a 23-protein inflammatory serum signature that distinguished AChR-antibody–positive MG patients from healthy controls, with cytokine- and inflammation-related markers such as CCL28, TNFSF14, 4E-BP1, TGF-*α*, and ST1A1 ranked among the leading candidates (Bhandage et al., 2024). Hussain et al. reported residual fibrinogen-related proteins as candidate biomarkers across different MG serotypes, supporting the presence of coagulation-related alterations in MG (Hussain et al., 2023). Chen et al. integrated large-scale human plasma proteomics with genetic instruments and identified MG-associated proteins at the population level, providing additional evidence that immune-inflammatory and complement-related protein alterations are relevant to MG risk (Chen et al., 2025). In addition, Yang et al. and Lin et al. focused on thymoma-associated MG; Yang et al. identified KRAS and SELP as serum proteins associated with docetaxel treatment response, whereas Lin et al. highlighted HLA-A, C5, and SELL as proteins associated with postoperative prognosis (Yang et al., 2023; Lin et al., 2025). These studies therefore mainly addressed inflammatory signatures, fibrinogen/coagulation-related markers, population-level risk-associated proteins, or thymoma-related treatment/prognostic contexts, rather than a baseline candidate serum protein panel in non-thymoma-selected AChR-positive MG. Compared with these published MG proteomic studies, the present work has several exploratory but distinctive features. First, our cohort focused on AChR-antibody–positive MG patients without recent corticosteroid, immunosuppressive, IVIG, or plasma exchange treatment, which may reduce treatment-related effects on serum protein abundance. Second, DIA-based serum proteomic screening was followed by PRM verification in the same discovery cohort and ELISA validation in an independent cohort, providing a stepwise candidate-prioritization strategy. Third, although the five prioritized proteins in our study—PROS1, PROC, C4BPA, PFN1, and TLN1—do not overlap at the individual-protein level with the leading candidates reported previously, the pathway-level signals converge with prior studies on immune inflammation and complement/coagulation regulation, while further extending the comparison to cytoskeleton/adhesion-related processes. In particular, PROS1, PROC, and C4BPA may reflect complement/coagulation regulatory responses, whereas PFN1 and TLN1 may reflect cytoskeleton- and adhesion-related alterations. Therefore, the novelty of our study does not lie in being the first to implicate complement or coagulation pathways in MG, but rather in integrating complement/coagulation regulators with cytoskeleton/adhesion-related proteins into an exploratory candidate serum protein panel for AChR-positive MG. Nevertheless, this proposed coagulation–complement–inflammation–cytoskeleton framework remains a pathway-level, hypothesis-generating model, and further functional experiments are required before causal mechanistic conclusions can be drawn.

Although the DIA discovery–PRM verification–ELISA validation strategy provides preliminary support for the differential abundance and exploratory diagnostic potential of these five candidate proteins, several limitations must be acknowledged. The discovery cohort size was relatively small, which may limit generalizability and increase the risk of unstable estimates or overfitting. In addition, the discovery-phase serum proteomics relied on Top 14 high-abundance protein depletion, an immunoaffinity-based step that can introduce coverage or co-depletion bias and potentially influence the relative estimates of certain protein families, including coagulation/complement-related proteins. MG is clinically heterogeneous, including differences in age at onset, thymic pathology, disease severity, and serological subtype. Because our cohorts included only anti-AChR antibody–positive patients and did not include MuSK-positive, LRP4-positive, or seronegative MG patients, the applicability of this panel to other serological subtypes remains uncertain. Another important limitation is the incomplete control of clinical and biological confounders. Although age and sex were comparable between groups, and patients receiving corticosteroids, immunosuppressants, IVIG, plasma exchange, or other MG-specific treatments within three months before sampling were excluded, serum proteomic profiles may still be influenced by MGFA class, disease duration, AChR antibody titers, systemic inflammatory status, BMI, comorbidities, and other biological factors. Because of the limited sample size, we did not perform MGFA-stratified analyses, severity–protein correlation analyses, or multivariable adjustment. Therefore, the observed protein differences should be interpreted as MG-associated exploratory findings rather than MG-specific biomarkers. Although we constructed an exploratory five-protein logistic regression model, the limited sample size prevented robust external validation. Therefore, the apparent AUC of the combined model may be inflated, and the LOOCV result should also be interpreted cautiously. Future studies with larger independent cohorts and disease-control populations are required to validate the diagnostic performance and generalizability of this five-protein panel. Although Benjamini–Hochberg FDR correction was applied to reduce the risk of false positives, the small discovery cohort and high-dimensional nature of the proteomic dataset still mean that some findings may be unstable. Together, these statistical and design constraints underscore the need for larger, multicenter cohorts—including MuSK-positive, LRP4-positive, and seronegative MG patients—combined with FDR-adjusted analyses, cross-validation, disease-control groups, and more comprehensive modeling to minimize bias and improve generalizability. Another important limitation is that the proposed coagulation–complement–inflammation–cytoskeleton framework was inferred from pathway enrichment, PPI network analysis, and protein-level validation, rather than directly demonstrated by functional experiments. Therefore, this model should be interpreted as a pathway-level, hypothesis-generating framework rather than an experimentally proven causal cascade. Future studies should examine complement deposition, MAC formation, coagulation/complement regulatory activity, and PFN1/TLN1-mediated cytoskeletal remodeling using Western blotting, immunofluorescence, complement activity assays, and relevant cellular or animal models of MG.

The robustness of our core findings is supported by two design features: (1) stringent statistical thresholds for biomarker selection at the discovery stage and (2) rigorous validation of the five-protein panel via PRM and ELISA in an independent cohort of 20 MG patients and 20 controls. Future studies should focus on (1) validating biomarker performance across clinically heterogeneous, multicenter cohorts; (2) exploring associations with clinical subtypes, disease activity, and treatment response; and (3) dissecting the crosstalk between complement/coagulation pathways and cytoskeletal regulation, with the aim of developing novel therapies targeting MAC formation or restoring NMJ stability.

## Conclusion

In summary, this study employed serum proteomics to systematically characterize differentially expressed proteins between patients with MG and healthy controls. We detected increased levels of PROS1, PROC, and C4BPA and decreased levels of PFN1 and TLN1 in MG. These proteins were mainly associated with pathway-level changes involving complement/coagulation regulation and actin cytoskeleton-related processes, rather than being regarded as direct evidence of a causal damage cascade. ROC curve analysis showed preliminary discriminatory performance in this exploratory cohort, and subsequent ELISA validation supported the differential abundance of these candidate proteins. Collectively, our results provide exploratory insights into circulating protein alterations in AChR-positive MG and suggest that PROS1, PROC, C4BPA, PFN1, and TLN1 may represent candidate serum biomarkers requiring further validation. Because of the limited sample size and the lack of functional mechanistic experiments, their diagnostic utility, disease specificity, and mechanistic roles remain to be confirmed in larger multicenter cohorts and experimental studies.

## Data Availability

The datasets presented in this study can be found in online repositories. The names of the repository/repositories and accession number(s) can be found in the article/Supplementary material.

## Footnotes

1
https://string-db.org
