# Supplementary material for: DIA-based serum proteomics of the complement–coagulation cascade identifies candidate biomarkers for myasthenia gravis
Source: Front Neurosci. 2026 Jul 13;20:1810549. doi: 10.3389/fnins.2026.1810549 (PMC13403498; doi:10.3389/fnins.2026.1810549)
Supplement: Supplementary file 4 [file Table_2.DOCX]

**Quantitative Analysis**

The raw LC-MS datasets were first searched against database and converted into matrices containing Normalized intensity of proteins. The Normalized intensity (*I*) was transformed to the relative quantitative value (*R*) after centralization. The formula is listed as follow where *i* represents sample and *j* represents protein:

*R_ij_ = I_ij_ / Mean(I_j_)*

**Differential Analysis**

Firstly, the samples to be compared were selected in pairwise groups, and the fold change (*FC*) was then calculated by the ratio of the mean intensity for each protein in two sample groups. For example, to calculate the fold change between sample A and sample B, the formula is shown as following: *R* denotes the relative quantitative value of the protein, *i* denotes the sample and *k* denotes the protein.

*FC_A/B,k_ = Mean(R_ik_, i∈A) / Mean(R_ik_, i∈B)*

To calculate the statistical significance of difference between groups, the student's T test was performed on the relative quantitative value of each protein from the two sample groups. *P* value < 0.05 was usually considered as the threshold for significance. Therefore the relative quantitative value of proteins was applied with log2 transformation typically. The formula is shown as following:

*P_ik_ = T.test(Log2(R_ik_, i∈A), Log2(R_ik_, i∈B))*

The protein with *P* value < 0.05, the fold change > 1.5 was regarded as significant up-regulated protein, while the protein with *P* value < 0.05, the fold change < 1/1.5 was regarded as significant down-regulated protein.

**Functional Enrichment**

Fisher’s exact test was used to analyze the significance of functional enrichment of differentially expressed proteins (using the identified protein as the background). Functional terms with Fold enrichment>1.5 and *P* value <0.05 were considered as significant.

**Enrichment-based Clustering**

To evaluate the functional similarities and distinctions among proteins with varying fold-change magnitudes, differentially expressed proteins were stratified into four distinct groups based on their expression fold changes, designated as Q1 through Q4. Cluster analysis based on functional enrichment was subsequently performed to systematically investigate their potential associations and distinct functional profiles across multiple annotation databases, including Gene Ontology (GO), KEGG pathways, Reactome, WikiPathways, and protein domains. Briefly, functional enrichment profiles were compiled across all four quadrants (Q1–Q4), and terms significantly enriched (*P* < 0.05) in at least one group were retained. The resulting filtered P value matrix underwent -log10 transformation to linearize enrichment significance. Hierarchical clustering analysis was then executed on the transformed dataset using Euclidean distance and the average linkage method to define clustering relationships. Finally, the enriched functional terms and their clustering topologies were visualized via a heatmap generated by the Heatmap function within the ComplexHeatmap R package.

**Protein-protein Interaction Network**

All differentially expressed protein database accession or sequence were searched against the STRING database for protein-protein interactions. Only interactions between the proteins belonging to the searched data set were selected, thereby excluding external candidates. STRING defines a metric called “confidence score” to define interaction confidence; we fetched all interactions that had a confidence score > 0.7 (high confidence). Interaction network form STRING was visualized in R package “visNetwork”.
